# De‐Mystifying the Clone‐Censor‐Weight Method for Causal Research Using Observational Data: A Primer for Cancer Researchers

**DOI:** 10.1002/cam4.70461

**Published:** 2024-12-06

**Authors:** Charles E. Gaber, Armen A. Ghazarian, Paula D. Strassle, Tatiane B. Ribeiro, Maribel Salas, Camille Maringe, Xabier Garcia‐Albeniz, Richard Wyss, Wei Du, Jennifer L. Lund

**Affiliations:** ^1^ Department of Pharmacy Systems, Outcomes, and Policy University of Illinois—Chicago Chicago Illinois USA; ^2^ Clinical Safety and Pharmacovigilance Daiichi Sankyo Inc., Basking Ridge New Jersey USA; ^3^ Division of Intramural Research National Institute of Minority Health and Health Disparities, National Institutes of Health Bethesda Maryland USA; ^4^ Department of Epidemiology School of Public Health, University of São Paulo São Paulo Brazil; ^5^ Center for Real‐World Effectiveness and Safety of Therapeutics (CREST) University of Pennsylvania Perelman School of Medicine Philadelphia Pennsylvania USA; ^6^ Inequalities in Cancer Outcomes Network London School of Hygiene and Tropical Medicine London UK; ^7^ RTI Health Solutions Barcelona Spain; ^8^ Division of Pharmacoepidemiology and Pharmacoeconomics Brigham and Women's Hospital, Harvard Medical School Boston Massachusetts USA; ^9^ School of Public Health, Southeast University Nanjing China; ^10^ Department of Epidemiology Gillings School of Global Public Health, University of North Carolina at Chapel Hill Chapel Hill North Carolina USA

**Keywords:** cancer screening, cancer treatment, causal inference, observational study, target trial emulation

## Abstract

**Background:**

Regulators and oncology healthcare providers are increasingly interested in using observational studies of real‐world data (RWD) to complement clinical evidence from randomized controlled trials for informed decision‐making. To generate valid evidence, RWD studies must be carefully designed to avoid systematic biases. The clone‐censor‐weight (CCW) method has been proposed to address immortal time and other time‐related biases.

**Methods:**

The objective of this manuscript is to de‐mystify the CCW method for cancer researchers by describing and presenting its core components in an accessible and digestible format, using visualizations and examples from cancer‐relevant studies. The CCW method has been applied in diverse settings, including investigations of the effects of surgery within a certain time after cancer diagnosis, the continuation of annual screening mammography, and chemotherapy duration on survival.

**Results:**

The method handles complex data wherein the treatment group to which an individual belongs is unknown at the start of follow‐up. The three steps of the CCW method involve cloning or duplicating the patient population and assigning one clone to each treatment strategy, artificially censoring the clones when their observed data are inconsistent with the assigned strategy and weighting the cloned and censored population to address selection bias created by the artificial censoring.

**Conclusions:**

The CCW method is a powerful tool for designing RWD studies in cancer that are free from time‐related biases and successfully, to the extent possible, emulate features of a randomized clinical trial.

## Graphical abstract

The clone‐censor‐weight (CCW) method has been proposed to address time‐related biases in causal research using observational data. The goal of this paper is to de‐mystify the CCW method through: (1) a narrative review of current applications in the cancer literature, (2) visual presentation and description of the CCW design and analytic components, (3) application of these visuals to several cancer case studies, and (4) discussion of important considerations and future directions.

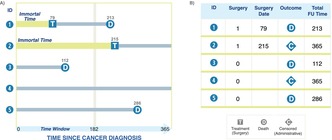

## 1 Introduction

Randomized controlled trials (RCTs) are the gold standard for establishing the efficacy of cancer therapeutics. However, it is not always feasible to implement an RCT due to ethical concerns, small eligible study populations, and high costs. Even when feasible, trial study populations often vary from real‐world populations in characteristics that limit generalizability. In such cases, observational studies may be informative to a variety of decision‐makers. In cancer research, healthcare providers rely on observational data to complement experimental evidence to make informed decisions [1]. Additionally, regulatory bodies (such as the US Food and Drug Administration and the European Medicines Agency) have recently published guidance on the inclusion of real‐world data in support of the assessment of medical products [2, 3].

The increased availability of large administrative and clinical databases, along with novel statistical methods, offers an opportunity to address gaps left by RCTs. However, to generate valid real‐world evidence, these data sources need to be used in a principled way to avoid systematic biases. Previous studies have shown that well‐designed observational studies can generate valid estimates of treatment effects similar to those of clinical trials [4, 5]. Specifically, target trial emulation [6] can be used to avoid some systematic biases by: (1) describing the components of a protocol for a hypothetical target trial and then (2) emulating each of these components using observational data.

The alignment of study eligibility, treatment assignment, and start of follow‐up—often referred to as time zero—is crucial for target trial emulation and prevents immortal‐time [7] (and other time‐related) biases. However, it can be challenging in observational studies when interventions cannot be identified at the point of study eligibility, but rather are observed over time. This is commonplace in cancer comparative effectiveness studies where interventions may include gaps between decision‐making and implementation (e.g., undergo surgery within 6 months from cancer diagnosis) [8], static time‐related strategies (e.g., continue annual mammography) [9], and dynamic treatment strategies (e.g., initiate androgen deprivation therapy when prostate‐specific antigen (PSA) test levels exceed a certain threshold) [10].

The clone‐censor‐weight (CCW) method has been proposed to address time‐related biases [11, 12]. Using this approach, researchers acknowledge that the treatment an individual will receive is unknown at the start of study follow‐up. To address this issue, the CCW method follows a three‐step process: (1) clones of each individual are created at the start of follow‐up and are assigned to each of the treatment strategies of interest; (2) clones are followed over time and artificially censored when their observed data deviates from the assigned treatment strategy; and (3) the study population is reweighted to account for the potential selection bias induced by the artificial censoring. Notably, the last two steps are shared with RCT analyses that aim to estimate the effect of treatment under complete adherence or the per‐protocol effect [13, 14]. While other methods such as landmark analysis and time‐dependent Cox models can also eliminate immortal time bias, they come with other drawbacks that the CCW approach avoids. Namely, landmark analyses can suffer from selection bias by analyzing only the sub‐population that survived to the landmark time. Time‐dependent Cox models estimate effects without grace periods, are difficult to apply for multi‐component treatments, and yield hazard ratios rather than risk contrasts.

To those unfamiliar, the implementation and interpretation of CCW can be challenging. Thus, the objective of this commentary—endorsed by the International Society for Pharmacoepidemiology (ISPE)–is to de‐mystify the CCW method through: (1) a narrative review of current applications in the cancer literature, (2) visual presentation and description of the CCW design and analytic components, (3) application of these visuals to several cancer case studies, and (4) discussion of important considerations and future directions. In addition, a glossary of commonly used terminology is provided.

## 2 Narrative Review of CCW Applications in Cancer

We first searched PubMed and EMBASE to identify observational studies published between January 2010 and February 2023 that applied the CCW method using two search strategies (detailed in Appendix 1). We then screened all resulting abstracts and titles to include only original research articles that appeared to focus on a cancer‐related research question. Twenty‐five articles were included for full‐text review, of which 7 were excluded because they did not apply the CCW method or were used in a context other than cancer. An additional five articles were “conceptual” in nature, largely explaining the value of the CCW method, but not focusing on its application to answer a specific research question. Finally, in Table S1, we extracted data from 13 articles that applied the CCW method to address a cancer‐relevant question. One reviewer (TBR) conducted a full text review and extracted relevant data. A second author (CM) reviewed the included studies and confirmed and modified, if necessary, the data extraction. Details of the articles excluded from the search are included in Tables S2 and S3.

These 13 articles spanned multiple tumor types using mostly North American datasets, with the majority (*n* = 10) being published since 2020, suggesting an increasing trend in the application of this method among cancer researchers. Populations of patients were well defined, and sometimes targeted groups of patients traditionally excluded from trials (e.g., older adults [10, 15, 16, 17] or with advanced cancer [18]). The interventions being compared varied from the receipt of a given treatment or test [10, 15, 16, 17, 18, 19] to the treatment setting (e.g., surgeon specialty or access to palliative care) [20, 21], duration [9, 22, 23], or timing [10, 24]. The main bias that researchers aimed to address by using the CCW approach was immortal‐time bias. Seven manuscripts included a comparison to “conventional” observational analyses of the data (e.g., a time‐varying Cox model): results often differed from those of the CCW, depending on the strengths of specific biases.

## 3 Description and Visual Presentation of the CCW Design and Analytic Components

This section aims to provide an overview and visual depiction of the main components of the CCW method. Suppose we are interested in estimating the effect of receiving surgery within 6 months (182 days) following a diagnosis of early‐stage lung cancer on 1‐year mortality among a population of older adults, compared with not receiving such surgery. This example was the focus of a paper applying the CCW method using data from the National Cancer Registry in England linked to administrative healthcare records [8]. To orient our visuals, we will include data from five hypothetical individuals as we work through the three steps of the CCW approach. We point the reader to the original reference [8] for a more comprehensive description of the technical details to apply the CCW method, including program code for implementation in Stata and R.

Prior to implementing the CCW method, several preliminary steps must be taken. One must clearly outline the hypothetical target trial that would be conducted to answer the specific research question. In Table 1, we specify the hypothetical target trial of the motivating example in early‐stage lung cancer, describing the following trial components: design, aim, eligibility criteria, treatment strategies, treatment assignment, treatment implementation, outcomes, follow‐up, censoring, adjustment variables, causal contrast, and estimands. This table then describes how each component of the hypothetical target trial can be emulated using observational data such as articulating the eligible study population (e.g., adults 70–89 years old diagnosed with early‐stage lung cancer) and identifying the timepoint(s) of eligibility (e.g., the date of lung cancer diagnosis). Anchoring the start of follow‐up, exposure assignment, and assessment of eligibility is a core feature of trial emulation using observational data [8, 25]. Then, one must explicitly define in detail the treatment strategies being compared, including which medical components (e.g., surgery) the treatment strategy will require and the timing of those components, hence defining a “grace period” (e.g., within 6 months or 182 days of lung cancer diagnosis). One must also decide whether an individual could meet eligibility multiple times during follow‐up, and what types of events (e.g., emerging toxicity or contraindications) would excuse an individual from receiving treatment while still being considered adherent to the protocol. The presence of ill‐defined interventions is problematic both for meeting causal inference assumptions and the statistical logistics of implementing the CCW method [12, 26, 27].

**TABLE 1:** Specification and emulation of a target trial of surgery versus no treatment within 6 months of diagnosis, elderly lung cancer patients diagnosed in 2012 in England.

Component	Target trial	Emulated trial using RWD
Design	Multicenter open‐label two‐parallel arm superiority randomized trial.	Cohort study
Aim	Estimate the effect of receiving surgery within 6 months of a NSCLC diagnosis on 1‐year overall survival	Same
Inclusion	Non‐Small Cell Lung Cancer patients diagnosed at age 70–89 years, at stage I or II, with a Charlson comorbidity index of 2 or less, a good performance status (≤ 2)	Same
Exclusion	Patients with a first major surgery in the month prior to diagnosis	Same
Treatment strategies	1. Major surgery within 6 months of diagnosis 2. No surgery in the 6 months after diagnosis	Same
Treatment assignment	Patients are randomly assigned to either strategy	Patients are non‐randomly assigned to a treatment strategy. Randomization is emulated via cloning of patients in both arms.
Treatment implementation	None	6‐month grace period
Outcome	Death from all causes within a year of diagnosis	Same
Type of outcome	Failure time	Same
Follow up	Follow up starts at diagnosis, equivalent to treatment assignment	Follow up starts at diagnosis, which does not correspond to treatment assignment
Censoring	Loss to follow up, administrative censoring	Loss to follow up, administrative censoring
Adjustment variables	Age at diagnosis (70–89 years), sex (1, 2), deprivation score (0–25), performance status (0, 1, 2), stage at diagnosis (1, 2), Charlson comorbidity index (0,1,2), emergency presentation (0,1)	Same
Causal contrast and analysis plan	*Intention‐to treat and per protocol effect* *Analysis:* Patients who deviate from the protocol are censored at time of deviation. A weighted Kaplan–Meier estimator was used to compute 1‐year risk differences	*Per protocol effect only* ^a^ *Analysis:* In each arm of the cloned data, patients who deviate from the protocol are censored at time of deviation. A weighted Kaplan–Meier estimator was used to compute 1‐year risk differences
Estimands	Differences in one‐year survival and restricted mean survival time at 1 year between arms	Same

^a^ An intention‐to‐treat (ITT) effect cannot be emulated because treatment intent was not available in the database and, at time zero, all participants adhered to both strategies and thus an ITT would not be informative.

**FIGURE 1:**
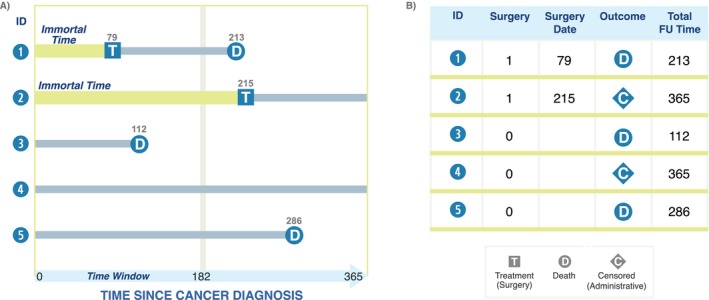
Visual depiction (A) and associated dataset (B) for the motivating example. In this example, older adults diagnosed with early‐stage lung cancer are followed for up to 365 days to assess the outcome of overall survival. The two treatment groups are surgery within 182 days of diagnosis and no surgery within 182 days of diagnosis. Patients 1 and 2 underwent surgery at some point during follow‐up; Patients 3–5 did not undergo surgery. Patients 1, 3, and 5 died within 365 days of diagnosis; Patients 2 and 4 were followed up until the end of the study (365 days) without an event.

An individual‐level study timeline and the associated raw data table for five hypothetical patients in the lung cancer observational study are pictured in Figure 1A,B. Note from Panel A, an analysis that starts follow‐up at cancer diagnosis but uses future surgery to define exposure groups at baseline (diagnosis) would lead to immortal time bias—where the effect of surgery on mortality is biased due to misclassification or exclusion of exposed person‐time [7]. In our example, because we are using a grace period of 182 days, the individual who died at Day 112 without surgery would have been classified into the no surgery group, regardless of whether they had been scheduled to undergo surgery, enriching this group with early deaths.

**FIGURE 2:**
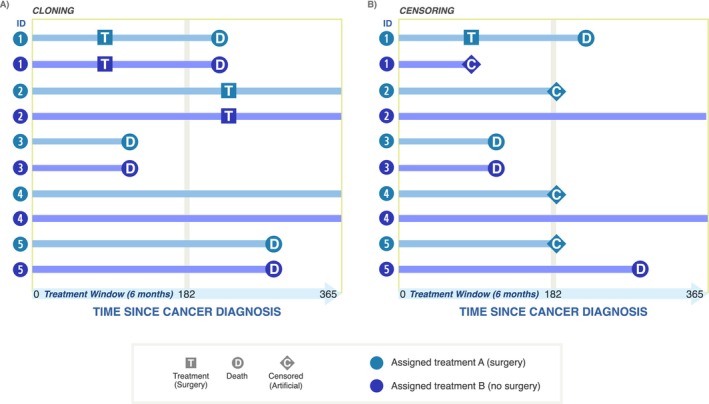
Visual depiction of the cloning and censoring components of the CCW method. When using the CCW method, patient data are duplicated, and clones are assigned to receive either treatment A (surgery within 182 days of diagnosis) or treatment B (no surgery within 182 days) (A). Data are reviewed and patients are then artificially censored when they deviate from their assigned treatment strategy (B). For example, the treatment B clone for Patient 1 is artificially censored when Patient 1 undergoes surgery within 182 days. The treatment A clone for Patient 2 is censored at the end of 182 days because it does not undergo surgery during this time window; however, the Patient 2 treatment B clone is followed until the end of the study period (even though they underwent surgery after 182 days, that is consistent with treatment B).

Considering the observational data available, the first step of the CCW method is to assign patients into exposure strategies according to their baseline data. Because baseline data can be compatible with more than one strategy, patients are assigned to *all* strategies with which their baseline data are compatible, effectively creating clones. In the lung cancer example, with two treatment strategies, this can be visualized as data from each individual being used twice and stacked on top of itself, as seen in Figure 2A. By “assign,” we mean create an exposure variable that takes on the value corresponding to treatment strategy A (i.e., underwent surgery within 182 days of diagnosis) for one entire set of clones—denoted in light blue in Panel A, and the value for treatment strategy B (i.e., did not undergo surgery within 182 days of diagnosis) for the other set of clones—denoted in dark blue in Panel B. Note that the crucial element of this step is that cloning is performed right at the start of follow‐up and is done without respect to any information on exposure status ascertained during follow‐up. As expected, after duplication, clones with treatment strategy A and treatment strategy B have the exact same baseline covariate profile (i.e., complete balance of measured confounders), because it is in fact the same underlying set of individuals.

The second step in the CCW method is to artificially censor the follow‐up of each clone at the timepoint when it first becomes apparent in the data that it is no longer consistent with its assigned treatment, as shown in Panel B. In conventional time‐to‐event observational studies, individuals are censored at loss‐to‐follow‐up or administrative end of study (“classic censoring”). In our simplified example, none of the five patients were censored due to these reasons. Classic censoring events can still occur and be handled in CCW analysis, but the censoring presently discussed is of a different variety: it is often referred to as “artificial censoring” because the analyst is imposing a censoring rule based on the treatment strategy under study and on the observed treatment data. When a clone deviates from the assigned treatment strategy, its follow‐up must be truncated. For instance, under treatment strategy A, “undergo surgical resection within 182 days of diagnosis,” if 182 days of data are observed and no surgery is performed for a particular individual, that individual's follow‐up time for the treatment strategy A clone must stop at 182 days (the end of the grace period to receive the intervention). It is at this time that we first become aware that the individual is not following treatment strategy A.

**FIGURE 3:**
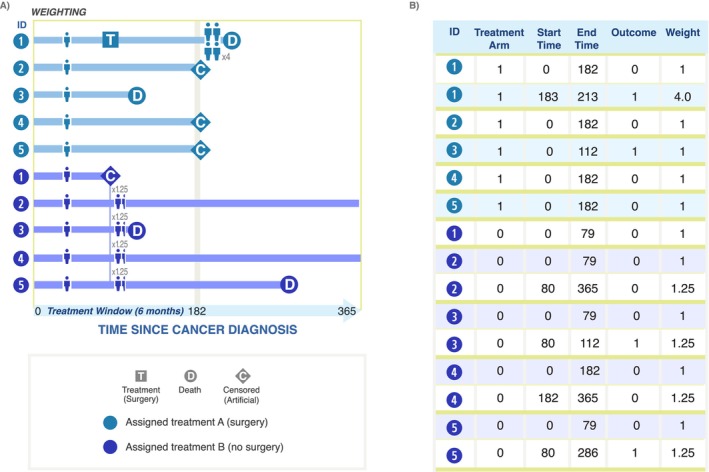
Visualization of the third component of the CCW method after hypothetical inverse probability weighting is applied (A) and the final analytic dataset (B). Panel A shows that for each treatment arm, compliant individuals (i.e., those who remain under follow‐up and not artificially censored) are upweighted to represent individuals in their treatment group who are artificially censored. Weighting can be implemented via inverse probability of treatment weighting in the original data (before duplication) or via inverse probability of censoring weighting in the duplicated data. For example, Patient 1 assigned to treatment A (surgery) is upweighted after Patients 2, 4, and 5 are artificially censored for not undergoing surgery within 182 days. Patients 2–5 assigned to treatment B (no surgery) are upweighted after Patient 1 undergoes surgery and deviates from the assigned treatment strategy. Panel B shows the final analytic dataset structure after applying the weights, with a new person‐time row for an individual clone anytime their weight changes. In this example, the impact of surgery within 182 days of diagnosis on 1‐year survival is being assessed. All individuals are followed for up to 1 year after diagnosis. Appendix 2 provides more detail about the weight calculations for a mock patient.

The third step in the CCW method is to weight the person‐time of uncensored individuals in each arm using inverse probability weighting (IPW), as depicted in Panel A of Figure 3A,B. After cloning, the baseline confounders are balanced. However, because the artificial censoring that occurs is not random, weights need to be used to address selection bias from factors that influence treatment receipt (and thereby artificial censoring) and the outcome of interest. IPW works by reweighting individuals over time according to their time‐varying confounders and censoring patterns (detailed further below). In the weighted population, assuming that the IPW model is correctly specified, treatment is independent of the measured confounders, eliminating post‐baseline confounding and selection bias due to those factors [13]. The artificial censoring in step two reflects the treatment selection observed among the study population, which is not random but rather a function of many variables that may also influence the outcome of interest. Models used to calculate IPW should include all prognostic baseline and post‐baseline variables that predict adherence to the assigned strategy.

In the literature, two approaches for implementing the calculation of inverse probability weights have been used. In one implementation, time‐varying inverse probability of *treatment* is calculated in the original eligible population (prior to cloning and censoring). This probability of treatment is then used to compute the weights (IPTW) in the resulting observations after cloning and censoring [9, 10, 12, 16]. The weighted outcome model is fit using the cloned, censored, and weighted observations. It is often a pooled logistic regression model predicting outcome occurrence at a given time, conditional on a flexible functional form for time, treatment strategy, and the interaction of time and treatment strategy. The coefficients of this model can be used to predict cumulative incidence over follow‐up. In another implementation, cloning and censoring are first applied and then the inverse probability of *censoring* is calculated in the resulting population [8, 15, 19, 21]. Weights are most often estimated using pooled logistic regression or Cox proportional hazards regression. A weighted outcome model follows, as described above for the IPTW implementation. With either IPW implementation, the time‐varying weights should remove the association between confounders and artificial censoring. IPWs can be stabilized and truncated to prevent the influence of extreme weights. In stabilization—using the IPCW implementation—the numerator of the weights is the probability of remaining uncensored conditional on baseline covariates. Truncating weights involves setting any extreme weights to a ceiling value (e.g., weights greater than the 99th percentile set to weight value of the 99th percentile). Further detail on the IPTW approach is included in Appendix 2.

A visualization of the final, analytic dataset is depicted in Panel B, which emphasizes the data structure for the analytic dataset with multiple rows per individual (one for each segment of follow‐up time) and time‐varying weights. Note that, the numeric values of the weights in the table are hypothetical, whereas an actual analysis would estimate these weights using information on several confounders.

Weighting aims to preserve the balance of covariates between treatment arms; whether this goal is achieved should be quantitatively assessed. Diagnostics for time‐varying confounding adjustment are emerging [28]. One simplified approach is to calculate standardized mean differences for evaluating covariate balance at the end of the specified grace period, as prior studies have done [8, 21]. However, this method is imperfect, as individuals who experience the event prior to the end of the grace period are not included in the balance metrics.

These three steps constitute the CCW method, to be performed ahead of the data analyses planned to estimate the effect of treatment on a given outcome. Any type of standard weighted estimator approach can be taken, such as weighted Kaplan–Meier curves that yield predicted probabilities of survival to a specified timepoint [29]. Importantly, bootstrapping or a robust variance estimator must be used when generating 95% confidence intervals to preserve and account for the correlation between clones of the same person. M‐estimation can alternatively be used.

**FIGURE 4:**
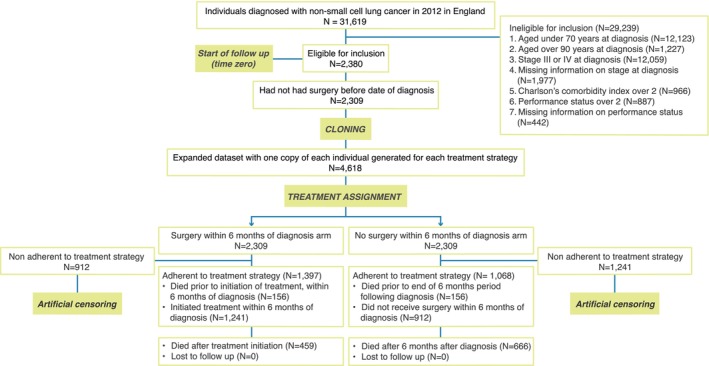
Study flow diagram reporting the core components of the CCQ method for the motivating example by Maringe et al.

A summary of all three steps of the CCW method can be reported using a flowchart (Figure 4) in which the elements relating to the selection of patients (e.g., eligibility criteria), their cloning in each of the arms considered, and their censoring are all clearly presented. We encourage researchers to use the target trial emulation shown in Table 1, the hypothetical patient‐level line diagrams shown in Figures 1, 2, 3, and the flowchart in Figure 4, to bring transparency to their study design and convey the steps of the CCW method to readers, increasing clarity and confidence in the approach.

## 4 Application of Visual Tools to Published Oncology Studies

The motivating example used in the prior section represents a single grace period application of the CCW approach. In the three examples below, we describe how prior studies comparing alternative cancer prevention or treatment strategies have used the CCW method in more complex settings. These examples illustrate a case with multiple grace periods for multi‐modal treatment, a static treatment strategy, and a dynamic treatment strategy—defined below.

## 5 Multiple Grace Periods: Definitive Chemoradiation Versus Trimodal Therapy in Esophageal Cancer

One of the major strengths of the CCW method is its ability to account for immortal time bias in research questions about multi‐modal treatments. For instance, a study used the CCW method to evaluate the effectiveness of trimodal therapy (chemoradiation and surgery) versus definitive chemoradiation (chemoradiation and no surgery) among older adults (66–79 years old) with locally advanced esophageal cancer [15]. In this example, because the treatment strategies included multiple, sequential intervention components, multiple grace periods, and in turn, multiple censoring criteria and multiple IPW models were needed to implement the CCW method. Follow‐up time for all patients began on the first day of chemotherapy treatment. Patients were then required to undergo radiation within 7 days (grace period 1); patients who did not receive radiation were censored in both treatment strategies. After the first grace period, whether or not the patient underwent surgery within 6 months was assessed (grace period 2). Those in the trimodal therapy treatment arm were censored if they did not undergo surgery within that time, and those in the definitive chemoradiation treatment arm were censored when they underwent surgery within the window. Corresponding patient timeline diagrams for five hypothetical individuals are included in Figure S1A,B.

This study used observational data from SEER‐Medicare linked data, from which treatment intention is unavailable, calling for the CCW approach. At the start of chemotherapy treatment, it was not known whether an individual planned to have radiation and surgery later. Additionally, due to the poor prognosis of locally advanced esophageal cancer, there was the possibility that patients who planned to undergo surgery would die before receiving the treatment, meaning the potential for immortal time bias would be present if treatment groups were defined at baseline using future surgery status.

### 5.1 Static Treatment Strategy: Continue Versus Stop Screening Mammography After Age 70

Another example of how the CCW method can be used is with a static treatment strategy, defined as a treatment that is delivered over time, but it is known at the onset when the treatment will be delivered (i.e., following a pre‐specified schedule) [30]. One example of a research question evaluating a static treatment strategy is whether continuing annual breast cancer screenings among women 70–84 years old reduces breast cancer mortality, compared to stopping screening [9]. For this study, treatment strategy A (annual screening mammography) consisted of receiving annual mammography throughout follow‐up (up to 8 years, in the absence of a breast cancer diagnosis), and treatment strategy B (stop screening) consisted of not receiving further screening. Both strategies allowed the receipt of a diagnostic mammogram at any time. In this application, cloning was necessary because all women were screened at baseline and therefore, for the first year of follow‐up (the next decision point for screening), were compliant with both strategies. To estimate the effect under full adherence, women in treatment strategy A (annual screening) were censored 14 months after the last screening if they had not received a subsequent one by then, and women in treatment strategy B (no screening strategy) were censored at the time they received a screening mammography.

Because of their interest in understanding the effects of age‐based screening strategies, the target trial process was repeated in 15 cohorts (one for each age from 70 to 84 years). Each woman could contribute to multiple cohort, if she met the cohort‐specific eligibility criteria, and per‐protocol analyses were conducted. Corresponding patient timeline diagrams for five hypothetical individuals are included in Figure S2A,B.

### 5.2 Dynamic Treatment Strategy: Timing of Androgen Deprivation Therapy Initiation in Prostate Cancer

A third example of how the CCW method can be used is with a dynamic treatment strategy, where the decision to undergo treatment is influenced by health status or disease progression, which can change over the course of follow‐up. For example, the comparative effectiveness of immediate initiation (treatment strategy A) versus deferring initiation of androgen deprivation therapy until disease progression (treatment strategy B) was studied among men with localized prostate cancer who experienced an asymptomatic PSA‐only relapse [10]. Immediate initiation was defined as starting androgen deprivation therapy within 3 months of PSA relapse. Deferred initiation was defined as starting androgen deprivation therapy within 3 months of disease progression (defined as the appearance of symptoms, metastases or a short PSA doubling time) or at least 2 years post‐PSA relapse. In this application, cloning was necessary because there could be patients who were compliant with both strategies for a period during follow‐up: those who had not initiated treatment in the first 3 months in the absence of a disease progression. They were compliant with the immediate treatment because there is a grace period of 3 months to start treatment, and also with the deferred treatment because they had not progressed. To estimate the effect under complete adherence, patient clones in treatment strategy A were censored 3 months after PSA relapse if they had not yet started treatment, and patient clones in treatment strategy B were censored 3 months after disease progression or 2 years after baseline if they had not yet started treatment. Because of the nature of these dynamic treatment strategies and the potential for treatment‐confounder feedback, no standard method to address time‐related biases, like landmark analysis or conventional regression, would have been able to answer this research question. Corresponding patient timeline diagrams for eight hypothetical patients are included in Figure S3A,B.

Other applications of the CCW method (including examples outside of cancer) are growing and incorporate study designs where there are more than two treatment arms [31], treatments with very short grace periods [18], and studies estimating the effect of treatment duration [11].

## 6 Discussion

Over the past 20 years, a robust literature has described the issue of time‐related biases in observational studies [1, 2]. Several analytic or design strategies for attributing person‐time and avoiding this bias exist, including the landmark method and time‐dependent Cox models. The advantages of the emerging CCW method over these traditional tools have been elaborated in several recent works [3, 4, 5]. Essentially, the CCW method allows a design and analysis that maps directly to a hypothetical target trial by aligning eligibility assessment, treatment assignment and the beginning of follow‐up, which additionally helps clarify the estimand(s) of interest. Additionally, it allows estimation of absolute measures of treatment effects, a preferred metric by patients and investigators [32], as well as adjustment of time‐varying confounding in the presence of treatment‐confounder feedback. Of note, cloning is more statistically efficient than randomly assigning individuals at baseline, an approach that also appropriately addresses the potential for immortal time when eligible individuals are compliant with more than one treatment strategy at baseline [9].

While this commentary provides an overview of the conceptual foundations for the CCW method and a visual description of its components, there are additional considerations required for robust implementation. First, clinical input is critical to ensure that the specified strategies reflect relevant clinical practice standards by specifying and incorporating contraindications into treatment strategy definitions (e.g., events that would excuse an individual from receiving treatment, but their time would still be considered adherent to the strategy) and by informing the length of the grace period that represents real‐world clinical practice. For example, in the screening mammography study described above [9], investigators specified that women who developed an incident cancer during follow‐up were excused from further screening in the “continue screening” arm, and in the esophageal cancer study above, the length of the first grace period was 7 days because delaying radiation beyond that time would not be standard practice, and the second grace period was 6 months because that is the usual timeframe to receive surgery for esophageal cancer. Second, for simplicity, we have disregarded the role of loss to follow‐up or “classic censoring” in our visualizations. If one believes that this form of censoring is due to factors that may also influence the outcome (e.g., moving out of the country or state), distinct inverse probability of (classical) censoring weights can be estimated to handle differential loss‐to‐follow‐up, provided such factors are accurately measured in the data. In practice, inverse probability weights can be computed and multiplied together to account for both classic and artificial censoring in the analysis. Third, we did not discuss the selection of time units for each of the specified example studies. Time can be discretized by various units (e.g., days, weeks, months) and the decision of the unit may be influenced by, for example, the length of the grace period and the precision of data available (e.g., exact dates versus month‐level reporting). Of note, if the goal is to use a pooled logistic model to approximate a hazard ratio, the risk of an event per unit of time must be small (< 10%) [14]. Finally, we have not included statistical software for implementation of the CCW method. While other research groups have made example code available for SAS, R, and STATA [8, 16, 19], further efforts should include the development of statistical packages that facilitate broader implementation of the CCW method and standardized tools for reporting methodological choices made while implementing CCW. These kinds of tools would enhance the transparency and reproducibility of studies implementing the method. We further encourage researchers to share their analytic code (e.g., R, Stata, SAS) on publicly available platforms (e.g., GitHub).

There are also important limitations of the CCW method that are worth noting. First, as with all observational studies, those implementing the CCW method are vulnerable to unmeasured or residual confounding. This limitation is inclusive of both baseline and time‐varying confounders that may be missing entirely or measured with error. Second, it is important to recognize that some treatment strategies, particularly those defined by “initiate intervention X prior to time *t*,” may be ill‐defined, as there are several possible ways to be adherent to a given strategy. In our initial example, one of the treatment strategies was to initiate surgery within 6 months (182 days) following diagnosis for early‐stage lung cancer. Individuals can be adherent to that strategy if they had surgery on Day 45 or Day 180 after diagnosis (among many other days). If the effect of the surgery depends on when it is initiated within this window, the interpretation of the effect estimate can be complicated and may require further clarification. For example, a further specification could be to initiate surgery within 6 months following diagnosis for early‐stage lung cancer at a uniform rate over the grace period. This issue may not be of major concern when grace periods are short, but further research is needed to describe and formalize these complex treatment effects. Finally, the CCW method can be quite sensitive to near violations of the positivity assumption, where certain combinations of confounding variables may lead to few individuals (of even none) remaining in a particular exposure group, rendering the weights to be large and unstable. In such instances, one may want to stabilize the weights or use outcome‐model focused approaches like g‐computation.

The application of the CCW method within the target trial emulation framework is gaining traction in epidemiologic circles. In this commentary, we sought to de‐mystify the CCW method for cancer researchers by describing and presenting its core components in an accessible and digestible format, leveraging visualizations and examples from cancer‐relevant studies. We hope that the visuals developed for this commentary assist cancer researchers in designing and communicating their own implementations of the CCW method that will further catalyze the dissemination of high‐quality and rigorous observational research using RWD.

## Author Contributions

**Charles E. Gaber:** conceptualization (equal), formal analysis (equal), methodology (equal), software (equal), visualization (equal), writing – original draft (lead), writing – review and editing (equal). **Armen A. Ghazarian:** formal analysis (supporting), investigation (supporting), methodology (supporting), writing – original draft (equal), writing – review and editing (supporting). **Paula D. Strassle:** conceptualization (supporting), formal analysis (supporting), methodology (supporting), visualization (equal), writing – original draft (supporting), writing – review and editing (supporting). **Tatiane B. Ribeiro:** data curation (equal), formal analysis (supporting), investigation (supporting), methodology (supporting), writing – original draft (equal), writing – review and editing (supporting). **Maribel Salas:** formal analysis (supporting), investigation (supporting), methodology (supporting), writing – original draft (supporting), writing – review and editing (supporting). **Camille Maringe:** conceptualization (supporting), formal analysis (supporting), methodology (supporting), visualization (supporting), writing – original draft (supporting), writing – review and editing (supporting). **Xabier Garcia‐Albeniz:** conceptualization (supporting), formal analysis (supporting), methodology (supporting), supervision (supporting), writing – original draft (supporting), writing – review and editing (supporting). **Richard Wyss:** formal analysis (supporting), investigation (supporting), writing – original draft (supporting), writing – review and editing (supporting). **Wei Du:** formal analysis (supporting), investigation (supporting), writing – review and editing (supporting). **Jennifer L. Lund:** conceptualization (lead), formal analysis (equal), funding acquisition (lead), investigation (equal), methodology (equal), project administration (lead), visualization (supporting), writing – original draft (equal), writing – review and editing (equal).

## Conflicts of Interest

Dr. Gaber receives academic salary support from an educational fellowship from pharmaceutical company AbbVie Inc., but the company does not sponsor this study. Dr. Lund reports salary support from the UNC Center for Pharmacoepidemiology (current members: GlaxoSmithKline, UCB BioSciences, Takeda, AbbVie, Boehringer Ingelheim, Astellas) and from other pharmaceutical companies to UNC (Roche, Janssen) for unrelated research projects. Maribel Salas and Armen Ghazarian are current employees of Daichi Sankyo Inc., but the company does not sponsor this study. Xabier Garcia de Albeniz is a full‐time employee of RTI Health Solutions. Tatiane Ribeiro is a current employee at Takeda, but the company does not sponsor this study.

## Precis

We introduce the clone‐censor‐weight (CCW) method for cancer researchers by describing and presenting its core components in an accessible and digestible format, using visualizations and examples from cancer‐relevant studies. The CCW method is a powerful tool for designing observational studies in cancer that are free from time‐related biases and successfully, to the extent possible, emulate features of a randomized clinical trial.

## Supporting information


Data S1.

## Data Availability

The authors have nothing to report.
