# Supplementary material for: De‐Mystifying the Clone‐Censor‐Weight Method for Causal Research Using Observational Data: A Primer for Cancer Researchers
Source: Cancer Med. 2024 Dec 6;13(23):e70461. doi: 10.1002/cam4.70461 (PMC11623977; doi:10.1002/cam4.70461)
Supplement: Supplementary file 1 — Data S1. [file CAM4-13-e70461-s001.docx]

**GLOSSARY**

**Artificial censoring =** Process of truncating an individual’s follow-up time when their treatment data is no longer consistent with the treatment strategy they were “assigned”; this censoring is distinct from censoring due to loss-to-follow-up or the administrative end of a study.

**Cloning =** Process of replicating an individual’s record in the dataset into as many copies as treatment strategies that their baseline data are compatible with. This is done to allow for the alignment of eligibility assessment, treatment assignment, and start of follow-up at time zero.

**Dynamic treatment strategy** = A treatment strategy that is sustained over time as opposed to a point-exposure (e.g., one-time surgery) but it may alter based on evolving conditions (e.g., treat with chemotherapy until a biomarker reaches a certain level).

**G-methods =** A set of analytic methods that can handle time-varying confounders that are influenced by prior treatment, including g-computation, g-estimation, and inverse probability weighted marginal structural models.

**Grace period =** Period of time during which a treatment strategy can be initiated.

**Immortal time bias** = Bias that occurs by using future information (usually pertaining to exposure) to define exposure groups at baseline; the time between baseline and this future information is guaranteed to be event-free (“immortal”) time.

**Inverse probability weighting =** A statistical method of up-weighting or down-weighting individuals based on their probability of something occurring (usually treatment). If successful, the process results in balance across groups with respect to the distribution of the factors contributing to the weights.

**Landmark analysis** = A method for addressing immortal time bias by selecting an exposure assessment window, classifying the exposure status of study population members according to their observed exposures during this window, and starting follow-up at the conclusion of the landmark window. This method excludes individuals who experience the outcome before the end of the window and ignores exposures that occur after the window.

**Propensity score** = The probability of receiving treatment (relative to a comparator treatment or no treatment), conditional on an individual’s history of selected covariates.

**Selection bias =** A bias introduced by conditioning on a common effect of two variables. In a directed acyclic graph, this is referred to as “conditioning on a collider”.

**Static treatment strategy =** A treatment strategy that is sustained over time (e.g., continue endocrine therapy use for three years), as opposed to a point-exposure (e.g., one-time surgery)—but it is defined at baseline and does not alter based on evolving conditions.

**Time zero =** The time for each individual when they meet eligibility criteria, are assigned to a treatment strategy, and their follow-up starts.

**Target trial emulation** = a framework for designing observational studies to mimic the features of randomized clinical trials as closely as possible. It involves specifying the hypothetical trial one wishes to emulate and mapping features of that trial to design and analysis choices in the corresponding observational study.

**APPENDIX**

**APPENDIX 1 – SEARCH STRATEGY**

Structured search in PubMed and EMBASE - Search date Feb 02, 2023

MEDLINE (via PubMed) - "clon*"[All Fields] AND "censo*"[All Fields] AND "weight*"[All Fields]) OR ("clone-censor-weight"[All Fields])

EMBASE - (clon* AND censor* AND weight* OR 'clone censor weight')

Supplemental Search: March 29, 2023

MEDLINE (via PubMed) - "trial*"[All Fields] AND "emulation*"[All Fields]) OR ("trial-emulation"[All Fields])

EMBASE - (trial* AND emulation* OR 'trial emulation')

MEDLINE (via PubMed) - "immortal*"[All Fields] AND "time*"[All Fields] AND "bias*"[All Fields]) OR ("immortal-time-bias"[All Fields])

EMBASE - (immortal* AND time* AND bias* OR 'immortal time bias')

**APPENDIX 2 – Detailed steps in inverse probability treatment weighting**

This is a toy example of how to compute weights to estimate the observational analogue of the per-protocol effect (using inverse probability of treatment weights). For illustration purposes, we focus on a mock patient assigned to the “No surgery within the six months after diagnosis” of the target trial described in **Table 1**.

Inverse probability of treatment weights are calculated as follows:

$$W_{t}^{A}=\prod_{k=0}^{t} \frac{1}{f(A_{k}\left| \overline{A}_{k-1}, \overline{L}_{k},\overline{Y}_{k-1}=0) \right.}$$

Informally, the denominator of this weight at time *k* is the probability of being adherent to the assigned treatment strategy.

Notes:

- *A_k_* represents the exposure (surgery) at time *k. f(A_k_)* is the probability density function of *A* at each month of follow-up, *k*. That is, for patients treated with *A* at month *k*, *f(A_k_)*=*P(A_k_*=*1)*; for patients not treated with *A* at month *k*, *f(A_k_)*=*P(A_k_*=0*)*=*1-P(A_k_*=*1)*
- The overbar indicates the history of a variable from the start of follow-up. $\overline{L}_{k}$and $\overline{Y}_{k-1}$are the covariate history (time-fixed (e.g., sex) and time-varying (e.g., frailty)) up to time k and outcome history up to time k-1, respectively.
- $P(A_{k}=1\left| \overline{A}_{k-1}, \overline{L}_{k},\overline{Y}_{k-1}=0) \right.$ is generally computed via a discrete hazards model, e.g., a pooled logistic regression. (Note: values included in the table below are hypothetical.)
- The symbol “Π” is the product notation and implies that subsequent terms are multiplied.
- The same process is followed for the other treatment strategy (i.e., “Surgery within six months”).

| ID | Assigned treatment strategy | *k* | *A_k_* | Adherent to the assigned treatment strategy? | $\boldsymbol{P}\mathbf{(}\boldsymbol{A}_{\boldsymbol{k}}\boldsymbol{=1}\left\vert{\overline{\boldsymbol{A}}}_{\boldsymbol{k}\mathbf{-1}}\mathbf{,}{\overline{\boldsymbol{L}}}_{\boldsymbol{k}}\mathbf{,}{\overline{\boldsymbol{Y}}}_{\boldsymbol{k}\mathbf{-1}}\mathbf{=0)} \right.$ | $\boldsymbol{f}\mathbf{(}\boldsymbol{A}_{\boldsymbol{k}}\left\vert{\overline{\boldsymbol{A}}}_{\boldsymbol{k}\mathbf{-1}}\mathbf{,}{\overline{\boldsymbol{L}}}_{\boldsymbol{k}}\mathbf{,}{\overline{\boldsymbol{Y}}}_{\boldsymbol{k}\mathbf{-1}}\mathbf{=0)} \right.$ | Factor for  $\boldsymbol{W}_{\boldsymbol{t}}^{\boldsymbol{A}}$ | $\boldsymbol{W}_{\boldsymbol{t}}^{\boldsymbol{A}}$ |
| --- | --- | --- | --- | --- | --- | --- | --- | --- |
| 1 | No surgery 6 months | 0 | No | Yes | 0.44 | 0.56 | $\frac{1}{0.56}$ | $\frac{1}{0.56}$ |
| 1 | No surgery 6 months | 1 | No | Yes | 0.29 | 0.71 | $\frac{1}{0.71}$ | $\frac{1}{0.56}\times\frac{1}{0.71}$ |
| 1 | No surgery 6 months | 2 | No | Yes | 0.40 | 0.60 | $\frac{1}{0.60}$ | $\frac{1}{0.56}\times\frac{1}{0.71}\times\frac{1}{0.60}$ |
| 1 | No surgery 6 months | 3 | No | Yes | 0.35 | 0.65 | $\frac{1}{0.65}$ | $\frac{1}{0.56}\times\frac{1}{0.71}\times\frac{1}{0.60}\times\frac{1}{0.65}$ |
| 1 | No surgery 6 months | 4 | No | Yes | 0.33 | 0.67 | $\frac{1}{0.67}$ | $\frac{1}{0.56}\times\frac{1}{0.71}\times\frac{1}{0.60}\times\frac{1}{0.65}\times\frac{1}{0.67}$ |
| 1 | No surgery 6 months | 5 | No | Yes | 0.28 | 0.72 | $\frac{1}{0.72}$ | $\frac{1}{0.56}\times\frac{1}{0.71}\times\frac{1}{0.60}\times\frac{1}{0.65}\times\frac{1}{0.67}\times\frac{1}{0.72}$ |
| 1 | No surgery 6 months | 6 | No | Yes | NA | NA | 1 | $\frac{1}{0.56}\times\frac{1}{0.71}\times\frac{1}{0.60}\times\frac{1}{0.65}\times\frac{1}{0.67}\times\frac{1}{0.72}\times1$ |

- The factor for weight, $\boldsymbol{W}_{\boldsymbol{t}}^{\boldsymbol{A}}$, is 1 at all k>5, as the “no surgery within six months” strategy does not require that individuals never undergo surgery after six months (e.g., an individual could get surgery in month 9 and still be adherent to the “no surgery within 6 months strategy”).
- Implementation of the Cox model or pooled logistic regression is up to the data settings and assumptions that the analyst is ready to make. The pooled logistic regression and the extended Cox model require data restructuring, with one record for each individual and time interval. Ultimately one expects that both models, if the effects of covariables are fitted appropriately, and time intervals are small enough, lead to almost identical weights.

**SUPPLEMENTAL MATERIALS**

**Supplemental Table 1. Applied oncology studies using the CCW method.**

| Author | Year | Cancer Type | Population | Intervention | Comparator | Primary Outcome | Reasons for cloning | Database | Geography |
| --- | --- | --- | --- | --- | --- | --- | --- | --- | --- |
| Boyne et al. | 2023 | Pancreas | Advanced pancreatic cancer | 5-fluorouracil, folinic acid, irinotecan, and oxaliplatin (FOLFIRINOX) | Gemcitabine plus nab-paclitaxel (GN) | Overall survival | Grace period | The Alberta Cancer Registry^a^ | Canada |
| Smith et al | 2022 | Prostate | Clinically localized prostate cancer | Initiate ADT within 3 months after PSA doubling time drops below a threshold | Do not initiate ADT below the threshold | Death from any cause | Dynamic treatment strategy | CaPSURE database^e^ | USA |
| Gaber et al. | 2022 | Esophagus | Locally Advanced Esophageal Cancer | Trimodality Therapy | Definitive Chemoradiation | Overall mortality, esophageal cancer–specific mortality, functional adverse events, and healthy days at home. | Grace period | SEER-Medicare | USA |
| Bryant et al. | 2022 | Lung | Stage III non-small cell lung cancer | Durvalumab for 12 months | Durvalumab for 9 months, Durvalumab for 6 months | Early treatment discontinuation, progression-free survival, and overall survival | Static time-related strategy | Department of Veterans Affairs (VA) Informatics and Computing Infrastructure (VINCI) | USA |
| Duchesneau et al. | 2022 | Breast | Metastatic breast cancer | Surgical resection | No resection | 3-year all-cause mortality | Grace period | National Cancer Database (NCDB) 2017 Participant Use File | USA |
| Weeks et al | 2022 | Ovary | Ovarian Cancer patients | Gynecologic Oncologist | Non-Gynecologic Oncologist | Chemotherapy initiation, receipt of cytoreductive surgery, 3-year all-cause survival, and 3-year cause-specific survival | Static time-related strategy | Iowa Cancer Registry (ICR) ^b^ | USA |
| Buranupakorn et al. | 2021 | Hepato-cellular Carcinoma | Hepatocellular carcinoma | Palliative Consultation | No palliative Consultation | Mean survival time (RMST) difference | Grace period | Electronic medical records of Chiang Rai Prachanukroh Hospital | Thailand |
| Boyne et al. | 2021 | Colon | Stage III colon cancer | 5-fluorouracil/leucovorin plus oxaliplatin (FOLFOX) | capecitabine plus oxaliplatin (CAPOX) chemotherapy, | Overall survival | Static time-related strategy | The Alberta Cancer Registry ^a^ | Canada |
| Garcia-Albeniz et al. | 2020 | Breast | Women eligible to breast cancer screening | Continue screening | Stop screening | Breast cancer mortality | Static time-related strategy | U.S. Medicare program, 2000 to 2008. | USA |
| Petito et al. | 2020 | Colon and rectum; Pancreas | Stage II colorectal cancer and advanced pancreatic adeno-carcinoma ^c^ | Initiate any dose of fluorouracil as first line treatment up to 3 months after post-surgery hospital discharge  Erlotinib added to gemcitabine within 12 weeks of gemcitabine initiation | No chemotherapy initiated within 3mo of post-surgery hospital discharge  No Erlotinib initiation within 12 weeks of gemcitabine initiation | Death | Grace period | SEER-Medicare | USA |
| Emilsson et al. | 2018 | Colon, Rectum, Breast, Prostate, Bladder | Stage I-III colon, rectum, breast, prostate, or bladder cancer | Initiate statin at any dose within 6 months after cancer diagnosis | Do not initiate statin therapy during follow-up | Cancer-specific mortality  All-cause mortality | Grace period | SEER-Medicare | USA |
| Garcia-Albeniz et al. | 2015 | Prostate | Prostate cancer ^d^ | Deferred androgen deprivation therapy: initiation at progression** or any time after 2 years since baseline, (continuous or intermittent). | Immediate androgen deprivation therapy: initiation (continuous or intermittent) at baseline | Death from any cause | Dynamic treatment strategy | CaPSURE database^e^ | USA |
| Garcia-Albeniz et al | 2014 | Colon | Localized colon cancer | Receiving at least one duplicate scan | Not receiving at least one duplicate scan | Mortality | Static time-related strategy | SEER-Medicare | USA |

^a^ (Population-level data from Alberta, Canada) linked with electronic medical records; ambulatory care services from the National Ambulatory Care Reporting System (NACRS) database, hospitalization Discharge Abstract Database (DAD), and the Practitioner Claims databases.

^b^ Iowa Cancer Registry (ICR) - collects statewide surveillance data and has been a member of the National Cancer Institute’s Surveillance, Epidemiology, and End Results (SEER) Program since 1973.

^C^ New diagnosis of stage II colorectal cancer and patients who received a new diagnosis of advanced pancreatic adenocarcinoma.

^d^ Prostate cancer treated with curative intention with a PSA-only relapse.

^e^ CaPSURE is a prostate cancer registry study of over 14,000 men with biopsy-proven prostate adenocarcinoma enrolled consecutively from over 45 community-based clinics, 3 academic institutions and 3 Veterans Administration hospitals since 1995

**Supplemental Table 2. Papers excluded due to not applying the CCW method or not focusing on cancer.**

| Author | Year | Cancer Type | Title |
| --- | --- | --- | --- |
| Reitblat et al. | 2021 | Prostate | Radical prostatectomy versus external beam radiation therapy for high-grade, clinically localized prostate cancer: Emulation of a target clinical trial |
| Heil et al. | 2023 | Colorectal | Improved Postoperative Outcomes after Prehabilitation for Colorectal Cancer Surgery in Older Patients: An Emulated Target Trial |
| Cain et al. | 2010 | HIV (non-cancer related), this was the first manuscript to discuss CCW method | When to Start Treatment? A Systematic Approach to the Comparison of Dynamic Regimes Using Observational Data |
| Kwee et al. | 2023 | Hepatocellular carcinoma (HCC) | Target Trial Emulation: A Design Tool for Cancer Clinical Trials |
| Dickerman et al. | 2020 | Colorectal Cancer | Emulating a target trial in case-control designs: an application to statins and colorectal cancer |
| Shen et al | 2017 | Prostate | Estimation of the optimal regime in treatment of prostate cancer recurrence from observational data using flexible weighting models. |
| Lazzati et al. | 2022 | Obesity-related Cancers | Effect of bariatric surgery on cancer risk: results from an emulated target trial using population-based data |

**Supplemental Table 3. Papers excluded due to conceptual focus.**

| Author | Year | Cancer Type | Title |
| --- | --- | --- | --- |
| Hernan et al. | 2018 | Prostate | How to estimate the effect of treatment duration on survival outcomes using observational data |
| Huitfeldt et al. | 2015 | Screening for Colorectal Cancer (Colonoscopy) | Methods to Estimate the Comparative Effectiveness of Clinical Strategies that Administer the Same Intervention at Different Times |
| Maringe et al. | 2020 | Lung | Reflection on modern methods: trial emulation in the presence of immortal-time bias. Assessing the benefit of major surgery for elderly lung cancer patients using observational data |
| Braitmaier et al. | 2022 | Breast | Effectiveness of Mammography Screening on Breast Cancer Mortality – A Study Protocol for Emulation of Target Trials Using German Health Claims Data |
| Kuehne et al. | 2022 | Ovarian Cancer | Causal analyses with target trial emulation for real-world evidence removed large self-inflicted biases: systematic bias assessment of ovarian cancer treatment effectiveness |


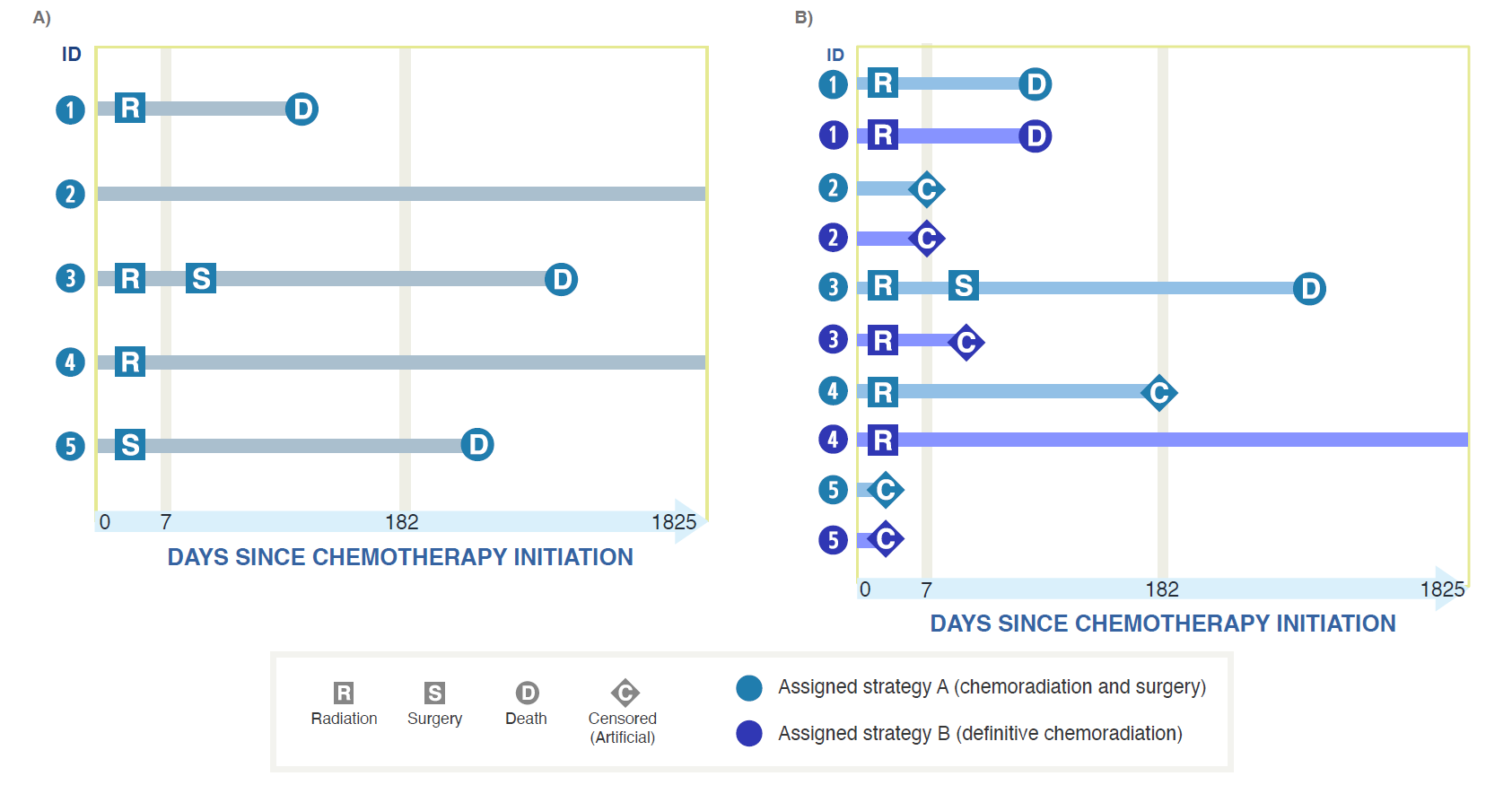


**Supplemental Figure 1A-B**. **Visualization of the CCW method for the esophageal cancer multimodal treatment strategy example.** Panel A depicts the original data patient timeline for five patients, and Panel B depicts their corresponding cloned and censored patient timeline data. The index date was the date of chemotherapy initiation. In strategy B, all patients must undergo radiation within seven days (grace period 1); patients who did not receive radiation were censored in both treatment strategies. For strategy A, patients had to undergo surgery within 182 days (grace period 2).


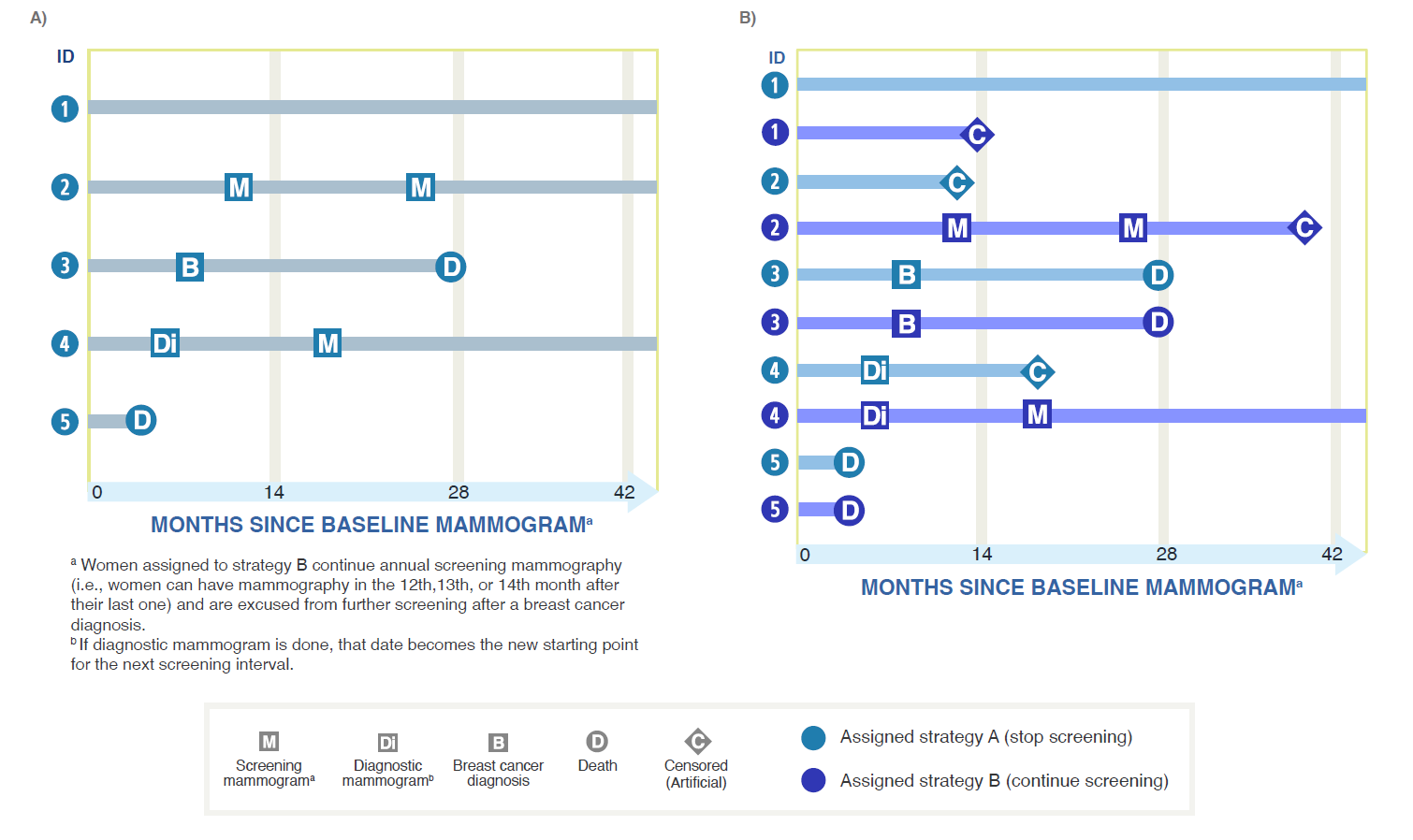


**Supplemental Figure 2A-B**. **Visualization of the CCW method for the mammography static treatment strategy example.** Panel A depicts the original data patient timeline for five patients (all age 70+ years at the time of their screening mammogram), and Panel B depicts their corresponding cloned and censored patient timeline data. In this paper, patients were included several times in a series of age-based cohorts, which is not depicted in this figure for simplicity. Women assigned to strategy A stop screening after their baseline exam. Women assigned to strategy B continue annual screening mammography (i.e., women can have mammography in the 12th, 13th, or 14th month after their last one) and are excused from further screening after a breast cancer diagnosis. If diagnostic mammogram is done, that date becomes the new starting point for the next screening interval.


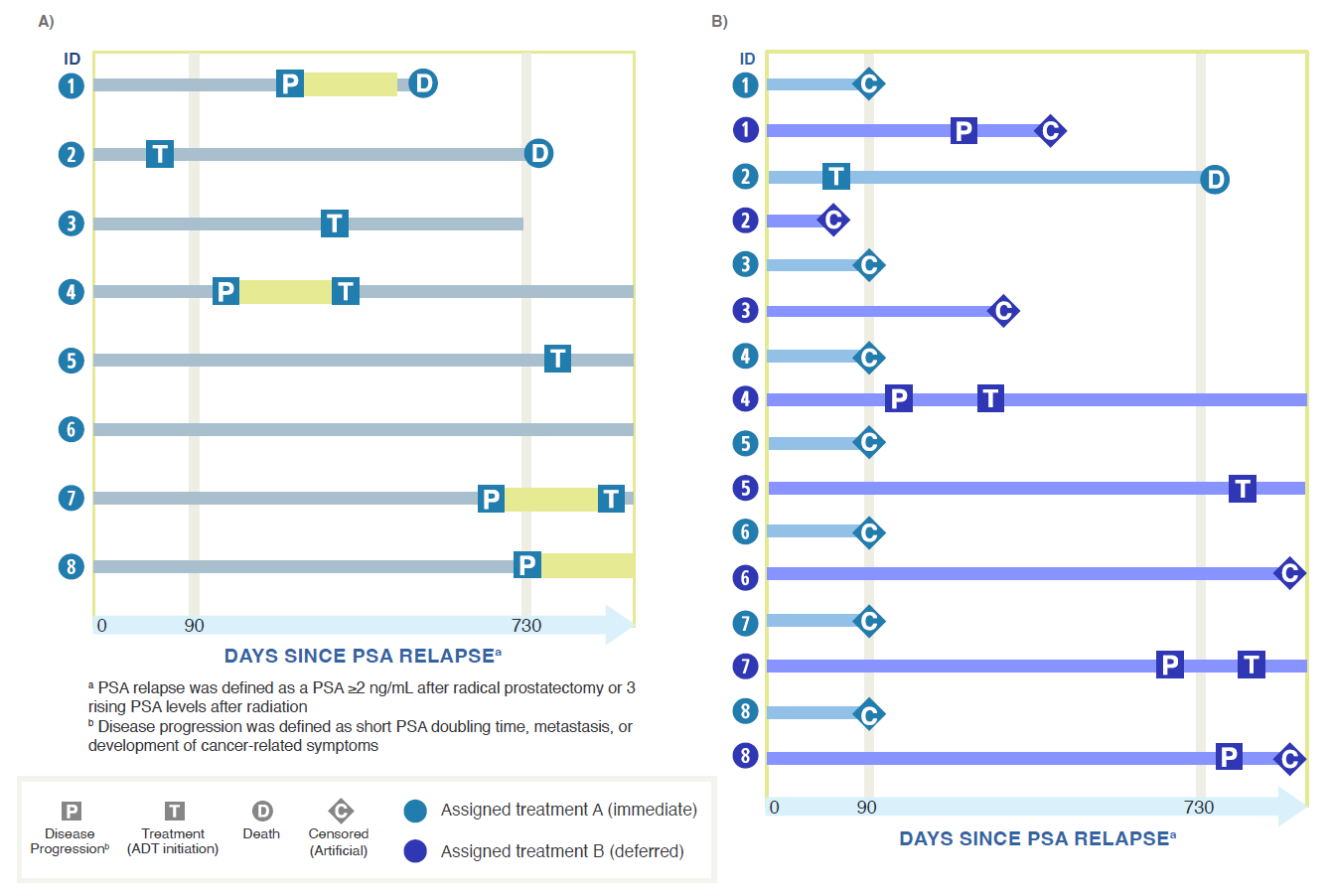


**Supplemental Figure 3A-B**. **Visualization of the CCW method for the dynamic treatment strategy using androgen deprivation therapy (ADT) initiation in men with PSA-only relapse after primary treatment (radical prostatectomy or radiotherapy) example.** Panel A depicts the original data patient timeline for eight hypothetical patients, and Panel B depicts their corresponding cloned and censored patient timeline data. Strategy A is immediate initiation (within 3 months of PSA relapse) of ADT and strategy B is deferred initiation (initiation within 3 months of disease progression or after more than 2 years after PSA relapse, regardless of progression).
